# Deactivation of the prefrontal cortex during exposure to pleasantly-charged emotional challenge

**DOI:** 10.1038/s41598-018-32752-0

**Published:** 2018-09-28

**Authors:** Kanji Matsukawa, Ryota Asahara, Miho Yoshikawa, Kana Endo

**Affiliations:** Department of Integrative Physiology, Graduate School of Biomedical and Health Sciences, Hiroshima University, 1-2-3 Kasumi, Minami-ku, Hiroshima 734–8551 Japan

## Abstract

Our laboratory reported that facial skin blood flow may serve as a sensitive tool to assess an emotional status and that both prefrontal oxygenation (as index of regional cerebral blood flow) and facial skin blood flow decrease during positively-charged emotional stimulation, without changing hand skin blood flow and arterial pressure. However, the focal location of the prefrontal responses in concentration of oxygenated haemoglobin (Oxy-Hb) that correlate with peripheral autonomic reaction remained unknown. This study was undertaken using 22-channel near-infrared spectroscopy to reveal spatial distribution of the responses in Oxy-Hb within the prefrontal cortex (PFC) during emotionally-charged audiovisual stimulation. Pleasantly-charged (comedy) stimulation caused a substantial decrease of Oxy-Hb in all regions of the PFC in 18 subjects, especially in the rostroventral frontopolar PFC, whereas negatively-charged (horror) or neutral stimulation (landscape) exhibited a weaker decrease or insignificant change in the prefrontal Oxy-Hb. In the rostral parts of the dorsolateral and ventral frontopolar PFC, the oxygenation response during comedy stimulation exhibited the most significant positive correlation with the decrease in facial skin blood flow. Thus the rostral regions of the PFC play a role in recognition and regulation of positive emotion and may be linked with neurally-mediated vasoconstriction of facial skin blood vessels.

## Introduction

The accumulating evidence suggests that the prefrontal cortex (PFC) is engaged in emotional recognition and processing as well as multiple functions such as attention, planning of motor act, and cognitive function. Lesion of the ventromedial prefrontal cortex and stroke of the right middle cerebral artery induced a deficit of emotional recognition^1,2^. Noninvasive stimulation with transcranial direct current of the ventromedial prefrontal cortex facilitated processing of pleasant scenes^3^. Thus the PFC may play an important role in creating emotional feeling. Neuroimaging studies for seeking neural correlates within the PFC have been conducted using positron emission tomography (PET) and functional magnetic resonance imaging (fMRI) in humans. Negatively-charged stimulation of sadness, fear, and disgust evoked an increase in activity of the medial, ventrolateral, and orbitofrontal PFC as well as the amygdala and anterior cingulate cortices^4–8^. In contrast, the prefrontal responses to positively-charged stimulation of pleasantness and happiness are controversial among the literatures. Regional cerebral blood flow (CBF) in the medial, dorsolateral (DLPFC), and orbitofrontal PFC was increased by positive emotional stimulation^6,9–12^. Other studies^4,13^ reported that regional CBF was decreased in the right PFC and amygdala, suggesting deactivation of the cortical and subcortical areas. Since the neuroimaging procedures accompany restriction of posture and body movement and probably impose stress more or less, such restriction may affect the emotional response, particularly to a positively-charged stimulation. As another problem of the imaging procedures, it is difficult to conduct real-time monitoring of prefrontal activity simultaneously with peripheral autonomic and circulatory changes, because the signal of blood oxygen level-dependent contrast has low time resolution and low signal to noise ratio.

The output of the PFC may affect the autonomic nervous system, because extensive connections from the prefrontal cortex to the autonomic nuclei or organs have been reported in the rat^14–16^ and monkey^17,18^. Chemical and electrical stimulation of the prefrontal cortex evoked the cardiovascular, respiratory, and metabolic changes in the rat^19–22^. Also, it is known that prefrontal activity correlated with the vagal component of heart rate variability during exercise^23–25^ and was involved in the behavioral and cardiovascular responses to fear conditioning^26^ and arousal stimulation^27^. Accordingly, it was hypothesized that if emotional stimulation may elicit modulation in prefrontal activity, a change in prefrontal activity may in turn evoke autonomic reaction. To test the hypothesis, two methodologies developed recently were taken into account. One was measurement of regional facial skin blood flow with laser speckle and/or Doppler flowmetry as a more sensitive tool to assess an emotional status, rather than limb skin blood flow and systemic haemodynamics^28^. The other was near-infrared spectroscopy (NIRS), which is able to detect the dynamic changes in concentration of regional oxygenated haemoglobin (Oxy-Hb) as index of cortical activity in a mobile condition. With two-channel NIRS probes attached on the forehead, we have recently revealed that positively-charged audiovisual stimulation can evoke a reduction in prefrontal oxygenation, which may imply a decrease in regional CBF and thereby may reflect reduced activity in the PFC^29^. However, since the spatial distribution of the Oxy-Hb response has not been examined, it was important for understating of neural mechanisms within the PFC to determine detail location of the prefrontal oxygenation response during emotionally-charged interventions (defined as audiovisual stimulation in this study). The present study was therefore undertaken (1) to measure the prefrontal Oxy-Hb responses on the different 22 locations using multichannel NIRS during emotionally-charged interventions and (2) to reveal the spatial distribution of the oxygenation responses in terms of both response magnitude and the extent of correlation with the changes in facial skin blood flow.

## Results

All data, including the prefrontal oxygenation, facial skin blood flow, cardiovascular variables, and subjective ratings, were compared among the three trials of each emotional intervention (comedy, horror, or landscape video), to verify the accuracy and reproducibility of the data. The individual data were thereafter averaged over the trials.

### Subjective ratings of pleasantness and consciousness

**Figure 1:**
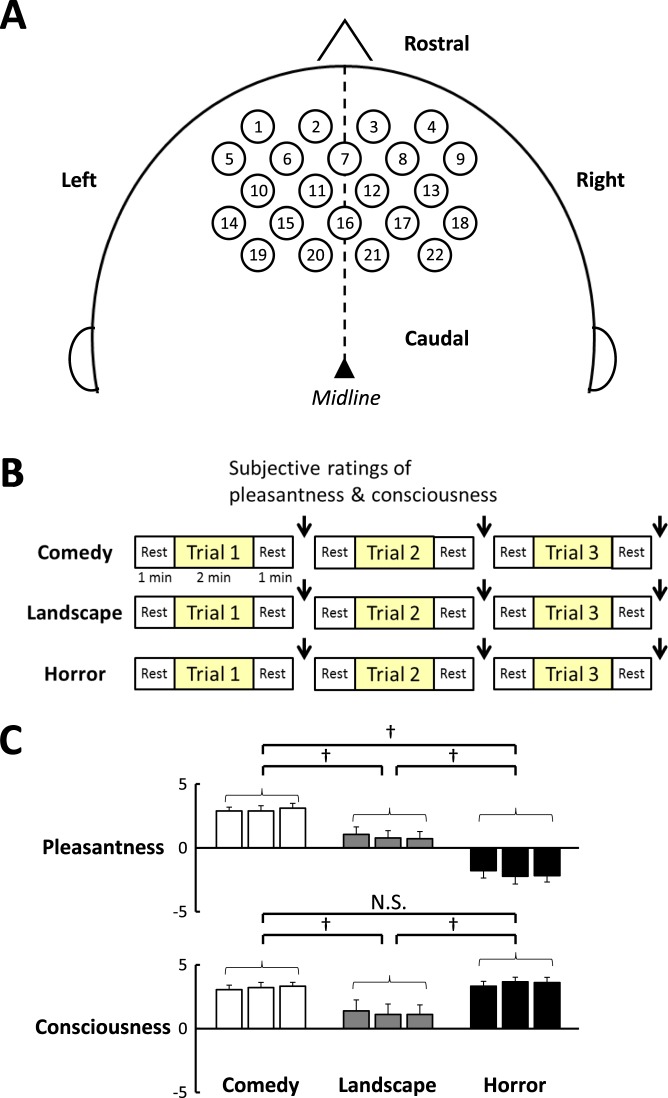
(**A**) Localisation of the 22 NIRS probed on the head. The anatomical locations of all NIRS probes, estimated by a three-dimensional digitizer, are summarised in Supplementary Figure S2. (**B**) experimental protocols about emotionally-charged audiovisual stimulation. The subjective ratings of pleasantness and consciousness were asked immediately after each trial of emotional stimulations as shown by arrows (↓). (**C**) comparisons of the subjective ratings of pleasantness and consciousness taken after each trial of emotional interventions (comedy, landscape, and horror). †, Significant difference (*P* < 0.05) among emotional interventions. N. S., not significant difference between emotional interventions.

As soon as every trial for an individual movie was completed, the subjective ratings of pleasantness and consciousness were asked against exposure to the emotionally-charged movie (as shown in Fig. 1). The subjective ratings of pleasantness and consciousness did not differ among the three trials of each emotional intervention (pleasantness, F_2,34_ = 3.019, *P* = 0.062 by two way repeated measures ANOVA; consciousness, F_2,34_ = 0.331, *P* = 0.720). The average pleasantness score was the highest with the comedy movie (2.6 ± 0.3) and the lowest with the horror movie (−2.3 ± 0.5); the difference in the pleasantness score was significant (F_2,34_ = 49.851, *P* < 0.001). On the other hand, the average consciousness scores with both comedy and horror movies (3.4 ± 0.4 and 3.7 ± 0.4, respectively) were higher as compared to the landscape movie (2.4 ± 0.5) (F_2,34_ = 19.639, *P* < 0.001). No correlation was detected between the extents of pleasantness and consciousness [correlation coefficient (*γ*) = −0.029, *P* = 0.714, n = 162 trials by Spearman’s rank correlation method].

### Cardiovascular responses during emotionally-charged stimulation

**Figure 2:**
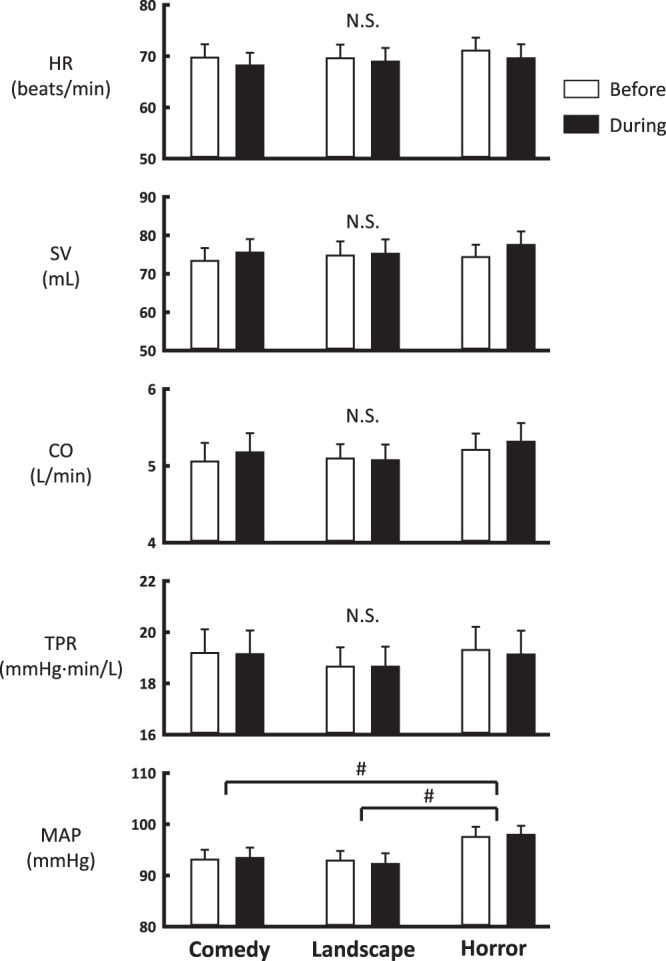
The average changes in heart rate (HR), stroke volume (SV), cardiac output (CO), total peripheral resistance (TPR), and mean arterial blood pressure (MAP) during an emotionally-charged intervention (comedy, landscape, and horror) in 18 subjects. The cardiovascular variables were analysed by two way repeated measures ANOVA with two main factors of time period (before vs. during) and emotional intervention. The cardiovascular variables were not significantly altered by the time period, suggesting no significant responses to a given emotional intervention. #, Significant difference (*P* < 0.05) between emotional interventions. N. S., not significant difference between emotional interventions.

Baseline heart rate (HR) and stroke volume (SV) were 70 ± 3 beats/min and 74 ± 3 mL, respectively; cardiac output (CO) was 5.1 ± 0.2 L/min accordingly. Baseline mean arterial blood pressure (MAP) and total peripheral resistance (TPR) were 94 ± 2 mmHg and 19 ± 1 mmHg∙min/L, respectively. Figure 2 summarises the cardiovascular responses during emotional interventions, which were analysed by two way repeated measures ANOVA with the factors of time period (before vs. during) and emotional intervention (comedy, landscape, and horror). Two way ANOVA revealed that HR and SV slightly changed during emotional stimulation (F_1,17_ = 7.946–26.438, *P* < 0.001 or *P* = 0.012) while CO, TPR, and MAP were not influenced (F_1,17_ = 0.002–2.958, *P* = 0.104–0.962), suggesting no substantial responses in the systemic haemodynamics to any emotional intervention. Furthermore, the cardiovascular variables, except MAP, did not differ among the emotional interventions (F_2,34_ = 0.090–1.394, *P* = 0.262–0.412). MAP was higher by 4–5 mmHg in horror intervention than comedy or landscape intervention (F_2,34_ = 10.038, *P* < 0.001). This difference was attributed to a rise in baseline MAP (Fig. 2).

### Prefrontal oxygenation during emotionally-charged stimulation

**Figure 3:**
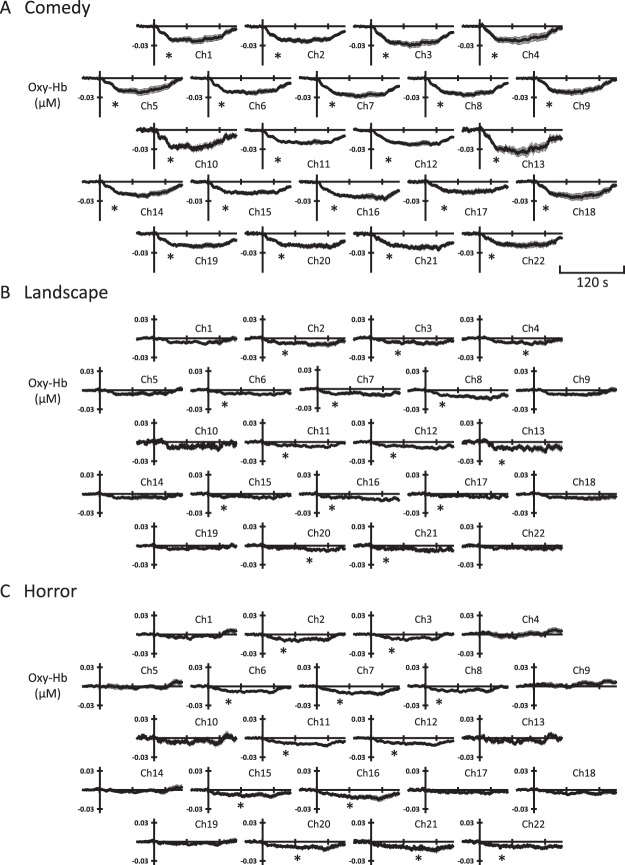
The time courses of the average changes in prefrontal oxygenated-haemoglobin (Oxy-Hb) over the 22 NIRS channels during each emotionally-charged intervention in 18 subjects. The time course data are shown as mean ± S. E. M. *, Significant response (*P* < 0.05) from the baseline before intervention. In addition, the symbol of (*) indicates the time point, at which the change in prefrontal Oxy-Hb became significant first in each tracing. The dynamic changes in concentrations of prefrontal deoxygenated-haemoglobin (Deoxy-Hb) during emotionally-charged challenges, as well as Oxy-Hb, are represented in Supplementary Figure S1.

Figure 3 represents the time courses of the Oxy-Hb changes in the PFC, which were averaged among the three trials of each emotional intervention and further averaged in 18 subjects. During exposure to the comedy movie, the Oxy-Hb at all 22 locations of the PFC decreased significantly (χ^2^-value = 1372.7–2100.2, *P* < 0.001 by Friedman repeated measures ANOVA on ranks). As shown in Fig. 3, immediately after the onset of the comedy stimulation, the prefrontal Oxy-Hb started to reduce and remained at a plateau level throughout the comedy stimulation. The latency of the significant reduction in Oxy-Hb was 29 ± 1 s. After the end of the comedy intervention, the prefrontal Oxy-Hb returned quickly to the baseline level. During exposure to either landscape or horror movie, the prefrontal Oxy-Hb decreased significantly at 55–64% of the 22 channels (mostly in the medial regions of the PFC) (χ^2^-value = 467.9–1066.0, *P* < 0.001). As compared to the Oxy-Hb decrease during the comedy intervention, the Oxy-Hb responses were blunted and delayed. The latency of the significant reduction in Oxy-Hb during viewing landscape or horror movie was 42 ± 6 s and 47 ± 4 s, respectively. On the other hand, the prefrontal Deoxy-Hb failed to change or slightly elevated in response to exposure to any emotionally-charged movie (as exemplified in Supplementary Figure S1).

**Figure 4:**
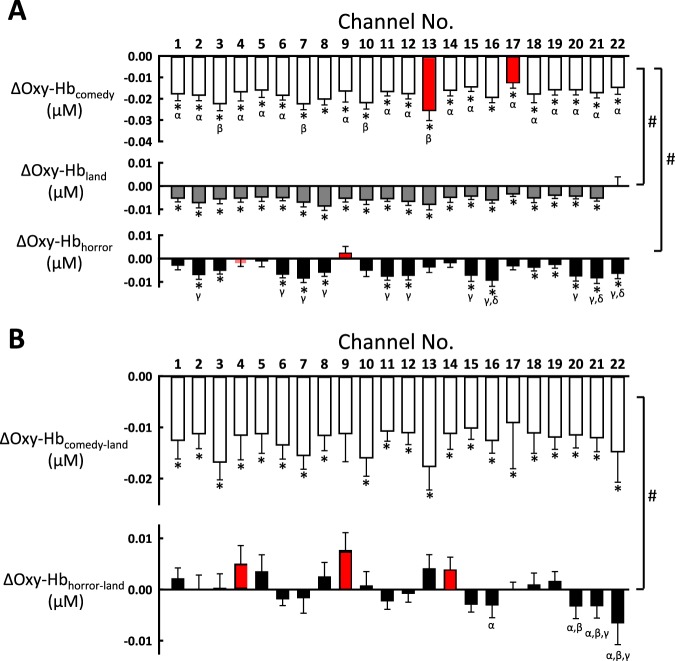
(**A**) The average changes in the Oxy-Hb responses during an emotionally-charged challenge (termed ΔOxy-Hb_comedy_, ΔOxy-Hb_land_, and ΔOxy-Hb_horror_) are summarised over the prefrontal 22 locations. The data were statistically analysed by two way repeated measures ANOVA with the factors of emotional intervention and prefrontal location. *, Significant difference (*P* < 0.05) in the Oxy-Hb response from the baseline. #, Significant differences (*P* < 0.05) in the Oxy-Hb response between emotional interventions. α, Significant differences (*P* < 0.05) from ΔOxy-Hb_comedy_ at the 13^th^ channel. β, Significant differences (*P* < 0.05) from ΔOxy-Hb_comedy_ at the 17^th^ channel. γ, Significant differences (*P* < 0.05) from ΔOxy-Hb_horror_ at the 9^th^ channel. δ, Significant differences (*P* < 0.05) from ΔOxy-Hb _horror_ at the 4^th^ channel. By assuming that landscape intervention corresponded to a neutral emotional status and involved non-emotional audiovisual stimulation, the Oxy-Hb responses during landscape intervention were subtracted from those during comedy or horror intervention (termed ΔOxy-Hb_comedy-land_ and ΔOxy-Hb_horror-land_, respectively). (**B**) the subtracted data were analysed by two way repeated measures ANOVA in the same manner as A. *, Significant difference (*P* < 0.05) in the Oxy-Hb response from the baseline. #, Significant differences (*P* < 0.05) in the Oxy-Hb response between emotional interventions. α, Significant differences (*P* < 0.05) from ΔOxy-Hb_horror-land_ at the 9^th^ channel. β, Significant differences (*P* < 0.05) from ΔOxy-Hb_horror-land_ at the 4^th^ channel. γ, Significant differences (*P* < 0.05) from ΔOxy-Hb_horror-land_ at the 14^th^ channel.

In Fig. 4, the Oxy-Hb responses during 2-min exposure of emotional interventions were analysed by two way repeated measures ANOVA with the factors of prefrontal 22-channel location and emotional intervention (comedy, horror, and landscape). The statistical analysis revealed significant main effects of both prefrontal location (F_21,336_ = 3.475, *P* < 0.001) and emotional intervention (F_2,32_ = 17.356, *P* < 0.001) and a significant interaction between them (F_42,667_ = 2.175, *P* < 0.001). At any prefrontal location, the Oxy-Hb was more decreased (*P* < 0.001 ~ *P* = 0.034) during comedy intervention as compared to the changes during horror and landscape interventions (Fig. 4A). On the other hand, the response in Oxy-Hb was not significantly different between horror and landscape interventions (*P* = 0.088–0.988) at all prefrontal locations (except the channels of 9 and 22, *P* = 0.026 or 0.041).

With the comedy intervention (Fig. 4A), the significant reduction of the Oxy-Hb (termed ΔOxy-Hb_comedy_) from the baseline was recognised (*P* < 0.001) at all prefrontal locations. The 13^th^ channel (corresponding to the right rostral DLPFC) demonstrated the greatest reduction, which was significantly greater than ΔOxy-Hb_comedy_ at the remaining prefrontal locations (*P* = 0.001–0.038, as denoted by α in Fig. 4A). The 17^th^ channel (corresponding to the right caudal DLPFC) showed the smallest reduction, which was significantly different from ΔOxy-Hb_comedy_ at several locations (*P* < 0.001 ~ *P* = 0.011, as denoted by β in Fig. 4A). With the horror intervention, the Oxy-Hb (termed ΔOxy-Hb_horror_) was significantly decreased (*P* < 0.001 ~ *P* = 0.017) from the baseline at 64% of the 22 locations. A significant difference in ΔOxy-Hb_horror_ was detected between the 9^th^ channel (corresponding to the right caudal DLPFC) and other 11 locations (*P* < 0.001 ~ *P* = 0.025, as denoted by γ in Fig. 4A) and between the 4^th^ channel (corresponding to the right frontopolar area) and other 3 locations (*P* = 0.002–0.044, as denoted by δ in Fig. 4A). However, with the landscape intervention, no significant differences in the Oxy-Hb response (termed ΔOxy-Hb_land_) were detected between any pair of the prefrontal locations, although ΔOxy-Hb_land_ was significantly decreased (*P* < 0.001 ~ *P* = 0.0364) from the baseline at all prefrontal locations (except the 22^th^ channel, *P* = 0.246).

By assuming that landscape intervention corresponded to a neutral emotional status (as judged by the results in Fig. 1C) and involved non-emotional audiovisual stimulation, the Oxy-Hb responses during landscape intervention were subtracted from those during comedy or horror intervention (Fig. 4B). When the subtracted data were analysed by two way repeated measures ANOVA with the factors of prefrontal location and emotional intervention, the analysis revealed statistical significance in the effects of emotional intervention (F_1,13_ = 12.693, *P* = 0.003) and interaction (F_21,273_ = 3.798, *P* < 0.001), but not prefrontal location (F_21,273_ = 1.338, *P* = 0.150). Accordingly, the subtracted Oxy-Hb responses (termed ΔOxy-Hb_comedy-land_ and ΔOxy-Hb_horror-land_, respectively) were significantly different between the two emotional interventions. With comedy intervention, ΔOxy-Hb_comedy-land_ was significant (*P* < 0.001 ~ *P* = 0.0135) as compared to the baseline at all prefrontal locations except the 9^th^ channel (*P* = 0.0595). However, ΔOxy-Hb_comedy-land_ did not differ between any pair of the locations (*P = *0.204–0.998). With horror intervention, although ΔOxy-Hb_horror-land_ did not change from the baseline at all prefrontal locations (*P* = 0.081–0.976), a significant difference in ΔOxy-Hb_horror-land_ (*P* = 0.010–0.045) was observed between the 4^th^, 9^th^, or 14^th^ channels and the most caudal DLPFC regions (the 16^th^ and 20–22^th^ channels).

### Relationships between prefrontal oxygenation and the changes in facial skin blood flow

**Figure 5:**
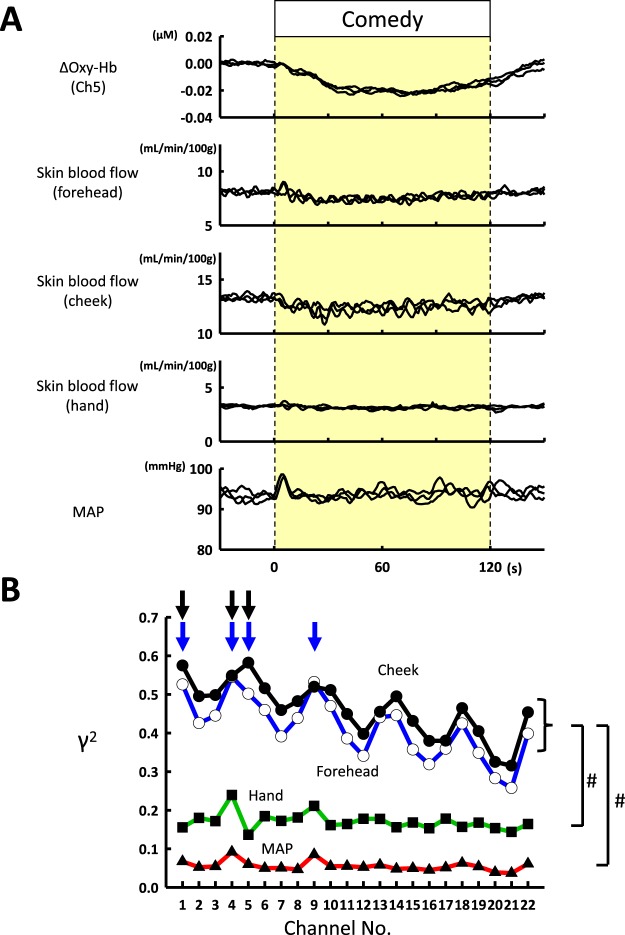
(**A**) The simultaneous changes in prefrontal Oxy-Hb (NIRS-channel 5), forehead and cheek skin blood flow, hand skin blood flow, and MAP during positively-charged comedy stimulation in 18 subjects. The tracings of the averaged time course data at the three trials of comedy stimulation are superimposed. (**B**) using the average time course data, the Spearman correlation analysis was conducted between the changes in skin blood flow (cheek, forehead, or hand) or MAP and the Oxy-Hb response obtained in a given NIRS channel. The square values (ϒ^2^) of the Spearman correlation coefficients are plotted against the NIRS channel number. The data (ϒ^2^) were statistically analysed by two way repeated measures ANOVA, having the main effect of the peripheral variables (skin blood flow and MAP) and prefrontal location. #, Significant difference (*P* < 0.05) among the peripheral variables (two way repeated measures ANOVA and posthoc Student-Newman-Keuls test). About forehead skin blood flow, the channels of 1, 4, 5, and 9 [indicated by blue arrows (↓)] had greater ϒ^2^ (*P* < 0.05) than the channels of 11, 12, 15–17, and 19–21; about cheek skin blood flow, the channels of 1, 4, and 5 [indicated by black arrows (↓)] had greater ϒ^2^ (*P* < 0.05) than the channels of 12, 15–17, and 19–21.

Skin blood flow in the forehead and cheek and the dorsum of the hand were simultaneously measured during exposures to the positively-charged comedy intervention. Figure 5A superimposes the time courses of the group-averaged responses in the prefrontal Oxy-Hb (at the NIRS channel 5), skin blood flow (forehead, cheek, and hand), and MAP during individual trials of comedy stimulation. Forehead and cheek skin blood flow decreased during comedy intervention (χ^2^-value = 816.797–843.080, *P* < 0.001), whereas hand skin blood flow and MAP did not change except the brief initial period for 10 s. To examine the spatial distribution of the prefrontal Oxy-Hb responses in terms of the extent of correlation with the peripheral circulatory variables, a multivariate correlation analysis was performed using the averaged time course data at every NIRS channel. The squares (*γ*^2^) of the Spearman’s rank correlation coefficients with skin blood flow and MAP are plotted over the 22 NIRS channels in Fig. 5B.

The correlation coefficient data of *γ*^2^ were analysed by two way repeated measures ANOVA with the factors of the peripheral circulatory variable (skin blood flow and MAP) and prefrontal location (as shown in Fig. 5B). The statistical analysis detected the significant effect of the peripheral variables (F_3,6_ = 23.214, *P* < 0.001). At any prefrontal location, the *γ*^2^ values with cheek and forehead skin blood flow were much greater (*P* = 0.036 ~ *P* < 0.001) than the *γ*^2^ values with hand skin blood flow and MAP, although the *γ*^2^ values did not differ between cheek and forehead skin blood flow (*P* = 0.270–0.966). In addition, hand skin blood flow and MAP exhibited no significant differences in the *γ*^2^ value at any NIRS channel (*P* = 0.054–0.292). The statistical analysis also revealed the significant effect of prefrontal location (F_21,42_ = 8.006, *P* < 0.001) and a significant interaction (F_63,126_ = 1.860, *P* = 0.002) between the main factors. In the prefrontal correlates with cheek skin blood flow, the greatest *γ*^2^ of 0.5823 occurred at the prefrontal 5^th^ channel (corresponding to the left rostral DLPFC); in the prefrontal correlates with forehead skin blood flow, the greatest *γ*^2^ of 0.5455 occurred at the 4^th^ channel (corresponding to the right rostroventral frontopolar). In correlation with both cheek and forehead skin blood flow, the rostral part of the PFC (at the NIRS channels of 1, 4, 5 and 9; as indicated by arrows in Fig. 5B) had a greater *γ*^2^ value (*P* = 0.001–0.045) than those in the caudal part of the PFC (at NIRS channels of 12, 15–17, 19–21).

### The changes in prefrontal oxygenation and facial skin blood flow during conversation

**Figure 6:**
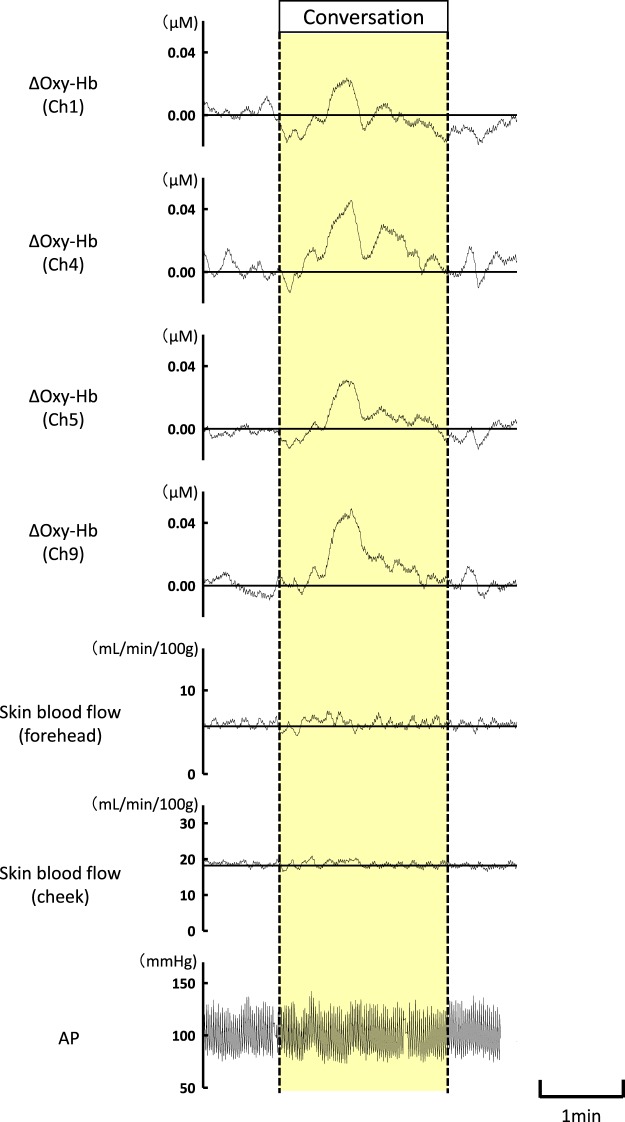
The simultaneous changes in prefrontal Oxy-Hb, forehead and cheek skin blood flow, and arterial blood pressure (AP) during conversation are represented in one subject. When conversation was made by asking the subjective ratings of pleasantness and consciousness, an increase in the prefrontal Oxy-Hb was observed on both sides, despite no change in the prefrontal Deoxy-Hb (not shown). Facial skin blood flow and AP did not represent substantial changes during conversation.

When conversation was made by asking the subjective ratings of pleasantness and consciousness, an increase in the prefrontal Oxy-Hb was observed on both sides (as exemplified in Fig. 6), without changing the prefrontal Deoxy-Hb. Forehead and cheek skin blood flow and arterial blood pressure (AP) usually failed to represent substantial changes during conversation.

## Discussion

We have reported that facial skin blood flow decreases during positively-charged emotional stimulation and may serve as a more sensitive tool to assess an emotional or mood status in humans^28^. Furthermore, we have revealed that the decrease in facial skin blood flow is closely correlated to the responses in prefrontal oxygenation measured with 2-channel NIRS probes attached on the forehead^29^. However, the spatial distribution of the dynamic changes in oxygenation over the PFC remained to be solved. The present study aimed to identify the foci of the oxygenation responses over the 22 locations of the PFC during emotionally-charged interventions and to examine to what extent prefrontal activity in each location was correlated with facial skin blood flow. The novel findings of this study are 1) that pleasantly-charged emotional intervention (comedy) decreased Oxy-Hb of all prefrontal regions, whereas negatively-charged stimulation (horror) and neutral stimulation (landscape) did not change significantly or slightly decreased the Oxy-Hb; 2) that when subtracting the Oxy-Hb response during neutral landscape stimulation from the Oxy-Hb response during comedy or horror stimulation, ΔOxy-Hb_comedy-land_ decreased below the baseline in all prefrontal regions, while ΔOxy-Hb_horror-land_ tended to increase in the right rostral DLPFC; 3) that the changes in prefrontal oxygenation (particularly in bilateral rostroventral frontopolar and rostral DLPFC) had a highly significant positive correlation with the changes in cheek and forehead skin blood flow, but not the changes in hand skin blood flow and MAP; 4) that, in contrast, prefrontal Deoxy-Hb did not change significantly or slightly increased during any of the emotional interventions. Thus the decrease in prefrontal oxygenation during the positively-charged emotional intervention suggests a reduction in regional CBF, which may reflect deactivation of the prefrontal cortex. Although the response size of prefrontal oxygenation could not be clearly differentiated among the regions within the PFC, correlation analysis with facial skin blood flow revealed that the bilateral rostroventral frontopolar and rostral DLPFC are the foci of the PFC responses.

The prefrontal response to positively-charged emotional stimulation of pleasantness and happiness is controversial among the previous studies with fMRI and PET, although it has been consistently reported that the medial [Brodmann’s area (BA) 9 and BA10], ventrolateral (BA47), and orbitofrontal (BA11) subdivisions of the PFC are activated during viewing unpleasant pictures or processing negative emotion^4–8^. Viewing pleasant pictures or processing positive emotion decreased regional CBF in the right PFC as well as the amygdala^4,13^, while the same kind of emotional stimulation increased regional CBF in the DLPFC (BA46), medial PFC (BA9 and BA10), and orbitofrontal PFC (BA11)^6,9–12^. The controversy may be at least partly due to a difference in the imaging methods. In addition, both imaging procedures need restriction of posture and body movement, which may affect the prefrontal response to a positively-charged emotional intervention more than a negatively-charged one. Instead, NIRS is considered useful as measurement of the dynamic changes in regional cerebral oxygenation in a less stressful mobile condition. Also, we have found that audiovisually-elicited emotional stimulation can elicit everyone more consistent pleasant feeling and subjective rating^28^, rather than in the case of viewing positively-charged images or facial expressions or recalling pleasant life episodes^30–33^. Using this intervention, the present study showed that the prefrontal Oxy-Hb decreased substantially during viewing a comedy movie, whereas it did not change significantly or slightly decreased during viewing horror and landscape movies. In contrast, prefrontal Deoxy-Hb did not change or slightly increased throughout any of the emotional interventions, suggesting that the changes in prefrontal oxygenation are attributed to the changes in regional CBF. Nevertheless, a possibility that the prefrontal oxygenation signal might involve extracranial skin blood flow within the illuminated tissue area cannot be neglected, because both prefrontal oxygenation signal and forehead skin blood flow decreased in parallel during viewing comedy movie. However, we found that prefrontal oxygenation was augmented during conversation without changing forehead skin blood flow (Fig. 6), in agreement with our previous study^29^. Such dissociation between the responses in prefrontal oxygenation and forehead skin blood flow has been recognised during a cognitive Stroop test and voluntary exercise^34–36^. Taken together, it is suggested that the prefrontal oxygenation signal recorded in this study may reflect prefrontal regional CBF, rather than extracranial skin blood flow. Furthermore, the changes in regional CBF are not passively evoked due to changes in perfusion pressure, because MAP did not alter during any of the emotional interventions (Figs 2 and 5).

Conversely, it is likely that a decrease in facial skin blood flow occurred in parallel to the reduction in prefrontal oxygenation during positively-charged emotional stimulation. In this study, the pleasantly-charged comedy stimulation caused a decrease in the prefrontal Oxy-Hb. This finding is supported by the previous result with PET by George *et al*.^4^, demonstrating that viewing pleasant pictures or processing positive emotion decreased regional CBF in the right PFC. Previous studies^4–8^ also reported that viewing unpleasant pictures or processing negative emotion activated the medial, ventrolateral, and orbitofrontal subdivisions of the PFC. However, we found that negatively-charged emotional stimulation (horror) failed to change substantially or decreased slightly oxygenation in all prefrontal locations, contrary to the previous findings^4–8^. The discrepancy about the prefrontal response to negatively-charged intervention may be explained by the fundamental limitation of NIRS that the prefrontal activity recorded is confined to the superficial cortical layer approximately 2 cm below the head surface. On the other hand, the Oxy-Hb_horror-land_ response, subtracted the data from horror to landscape stimulation, tended to increase the Oxy-Hb in the right rostral DLPFC (Fig. 4B). Taken together, it is of interest that activity of the right rostral DLPFC tended to show reversible responses to the emotional interventions, i.e., a decrease in activity during positive-charged stimulation and an increase in activity during negatively-charged stimulation.

It is difficult to identify an emotional or mood status in terms of the changes in systemic cardiovascular variables and limb skin blood flow, unless fear-induced emotional stress or a stress-induced defensive behaviour is elicited^37–42^. Instead, we have proposed facial skin blood flow as a better physiological candidate for estimating an emotional or mood status^28,29^. Facial skin blood flow decreased during viewing a positively-charged movie but they failed to exhibit a significant response to negatively-charged emotional stimulation^28,29^. In this study, we found a highly significant positive correlation between the decreases in prefrontal oxygenation and facial skin blood flow in the cheek and forehead during positively-charged emotional stimulation (Fig. 5). On the other hand, the decrease in prefrontal oxygenation had no significant relationships with the changes in hand skin blood flow and MAP. Based on the relationships, it is speculated that autonomic regulation of cutaneous blood vessels is different between the facial and limb regions and that the response in facial skin blood flow may occur in parallel with the response in prefrontal activity, at least during positively-charged emotional stimulation.

As far as we know, the efferent connections between the PFC and facial cutaneous blood vessels remain to be studied. Previous neurotracing studies have revealed extensive connections from the prefrontal cortex (especially the ventromedial subdivision) to the autonomic nuclei or organs in the rat and monkey^14–18^. Chemical and electrical stimulation of the prefrontal cortex evokes cardiovascular, respiratory, and metabolic changes^19–22^. Accordingly, if activity of the prefrontal cortex is altered by emotional intervention, it is possible that a change in prefrontal activity may in turn elicit an autonomic reaction. We preliminary found that electrical stimulation of the prelimbic area of the medial PFC decreased lip skin blood flow in the anaesthetised rat, while stimulation of the infralimbic area of the medial PFC increased lip skin blood flow without changing MAP (Matsukawa and Asahara, *unpublished observation*). Thus activation of the infralimbic prefrontal cortex may elicit facial skin vasodilatation at least in the rat. Although this idea is supported by the present finding that the decrease in facial skin blood flow may occur in parallel with the decrease in prefrontal activity, the direct cause-effect relationship between prefrontal activity and facial skin blood flow remains to be solved in humans.

Some substantial limitations are involved in this study. First, one important technical limitation of NIRS is that the oxygenation signal is confined to the superficial cortical layers of the PFC. Accordingly, the ventral parts of the PFC (such as the ventrolateral and orbitofrontal PFC) could not be approached with NIRS, because the subdivisions lie deep from the frontal surface of the head. Another technical limitation is a low spatial resolution as estimated from the distance of 3 cm between photoemitter and photodetector probes. Second, even if the change in regional CBF may reflect mass neural activity in a local brain region^43^, there is no way of knowing whether the increase in regional CBF involves excitatory or inhibitory neuronal activation or both. Third, although the movies of the comedy and landscape provided relatively stationary stimulation over the elapsed time period, the horror movie consisted of many variant scenes, which might induce not only fear and negative feeling but also neutral feeling. By this reason, the responses in prefrontal oxygenation became variable among subjects and even in a given subject. To avoid the nonstationary effect of the horror movie, use of negatively-charged static pictures taken from the database of the international affective picture system^44^ will be taken into account in future.

In conclusion, we have for the first time revealed that in the rostroventral frontopolar PFC and rostral DLPFC, pleasantly-charged emotional stimulation caused a decrease in Oxy-Hb, which had a good positive correlation with the changes in cheek and forehead skin blood flow. Furthermore, the Oxy-Hb in the right rostral DLPFC tended to represent the reversible responses to the emotional interventions (i.e., a decrease during positively-charged stimulation and an increase during negatively-charged stimulation). Based on the evidence, it is speculated that activity in the rostroventral frontopolar and rostral DLPFC plays a role in emotional recognition and processing.

## Methods

### Participants

Eighteen healthy volunteers (11 male and 7 female subjects) participated in the present study (age, 24 ± 1 years; body weight, 61 ± 3 kg; height, 165 ± 2 cm). None of the subjects suffered from any known cardiovascular and neuromuscular diseases and took any medication. All procedures and protocols in this study were performed in accordance with the 1964 Helsinki declaration and its later amendments or comparable ethical standards and were approved by the Institutional Ethical Committee of Hiroshima University (permit No. 1425). Informed consent from all individual participants was obtained prior to the experiments. All experiments were performed in thermoneutral and soundproof environment (room temperature, 25 ± 0.3 °C; relative humidity, 49 ± 2%).

### Subjective ratings of feelings stimulated by emotionally-charged movies

The subjective feelings of pleasantness and consciousness were asked immediately after each trial of the emotionally-charged stimulation, according to previous studies^28,45,46^. Pleasantness was rated with 11 grades from “the most pleasant” (+5) to “the most unpleasant” (−5). Consciousness was also rated with 11 grades from “the most conscious” (+5) to “the most unconscious” (−5). We explained to the subjects that “the most conscious” means the most awake condition being fully aware of the situation, while “the most unconscious” means the drowsiest or sleepiest condition being fully unaware of the situation. The assessment tables were displayed on a 60 inch TV display (Sharp Co., Tokyo, Japan) and the ratings were determined according to the feelings by individual subjects.

### Measurements of facial and limb skin blood flow and systemic cardiovascular variables

Forehead, cheek, and hand skin blood flow were recorded with laser Doppler flowmetry as reported previously^29^. Doppler flow probes were placed on the middle of the forehead, the left cheek, and the dorsum of the right hand. Electrocardiogram (ECG) and respiratory movement were monitored with a telemetry system (DynaScope DS-3140, Fukuda Denshi, Tokyo, Japan). AP was noninvasively measured with a Finometer (Finapres Medical Systems BV, Arnhem, the Netherlands), whose cuff was attached to the left middle or index finger. The beat-to-beat values of MAP, CO, SV, and TPR were calculated from the aortic pressure waveform by using a Modelflow® software as reported previously^28,29^. The cardiovascular variables and Doppler skin blood flow were stored to computers at a sampling frequency of 1 kHz as reported previously^29^.

### Oxygenated- and deoxygenated-hemoglobin concentrations of the PFC

The relative concentrations of the Oxy- and Deoxy-Hb of the bilateral prefrontal cortices were measured with a multichannel NIRS (FOIRE-3000, Shimadzu Co., Kyoto, Japan). The concentrations of the Oxy- and Deoxy-Hb at 22 sites in a 15 × 15 cm area were measured with the NIRS probes placed over the frontal surface of the head (referring to the international EEG 10–20 system) as shown in Fig. 1. The probes were mounted on a plastic helmet that was securely held by adjusting screws and straps over the subject’s scalp. The interprobe distance was 3 cm. Near-infrared light from three laser photodiodes with different wavelengths (775, 810, and 850 nm) penetrated brain tissue. Some of the light was absorbed by Hb and the remaining light scattered by the brain tissue was picked up with photodetectors. According to a mathematical formula developed by Matcher *et al*.^47^, the measured changes in light attenuation at the three wavelengths were converted into corresponding concentration changes for the Oxy- and Deoxy-Hb with a specially-designed programme for the FOIRE-3000. Since the oxygenation signal of NIRS is determined by a balance between oxygen supply and utilization in microcirculation, the Oxy-Hb signal does not directly monitor regional blood flow. If the Deoxy-Hb of the prefrontal areas remains at or near the baseline level throughout the interventions, the change in Oxy-Hb reflects the change in regional tissue blood flow, which may partly follow neural activity in the brain^48–52^. Another point to be considered is that the signals of the NIRS are influenced not only by regional CBF but also by extracranial blood flow within the illuminated tissue area. To clarify the possible contribution of skin blood flow to the Oxy- and Deoxy-Hb signals, forehead skin blood flow was simultaneously monitored.

The three-dimensional location of each NIRS probe was determined using a magnetic space digitizer (FASTRAK, Polhemus, Colchester, VT). Using a probabilistic registration method (NIRS-SPM)^53^, the position of each NIRS channel was identified on the Montreal Neurological Institute standard template^54^. A MRIcro programme^55^ probabilistically estimated the anatomical brain regions and BA corresponding to the location of a NIRS probe as we reported recently^56^ (as shown in Supplementary Figure S2).

### Experimental protocols

Audiovisual stimulation by emotionally-charged movies was controlled by a computer and displayed on the TV display with earphones approximately 2 m apart from the subjects, who sat on a comfortable chair. Three kinds of movies (comedy, horror, and landscape) were selected as emotionally-charged stimulation as reported previously^28,29^. Each of the emotionally-charged interventions (comedy, landscape, and horror) consisted of three consecutive trials involving different scenes captured from an individual movie. The comedy movie charged pleasant feeling, while the horror movie charged unpleasant one; the landscape movie induced neutral feeling (Fig. 1).

The prefrontal NIRS, cardiovascular, and facial and hand skin blood flow data were sampled at each trial, which contained the periods before (for 0.5 min), during (for 2 min), and after movie stimulation (for 0.5 min). At the pre- and post-stimulation periods, the subjects watched a small white circle on the TV black background. The data for 30 s before emotional stimulation were regarded as the baseline. Immediately after the cessation of each trial, the subjective ratings of pleasantness and consciousness were asked. The order of the three movie interventions was randomized. A time interval between trials in a given emotional intervention and between emotional interventions (i. e., a transit time from comedy to landscape) was approximately 4–5 min. The subjects were asked not to evoke any facial movement during emotional challenges as much as possible, because any skin movement due to facial expressions (such as smiling and laughing etc.) caused an artefact in laser Doppler blood flow measurements. Whenever such facial movement occurred during a trial of the emotional challenges, an artefact was recognised on the laser Doppler flow data and removed from the data acquired. Actually, a spike-like artefact in forehead skin blood flow was observed 4 times in 162 trials, while no visible artefact was observed in cheek skin blood flow. The mean duration of the artefacts in forehead skin blood flow was 0.4 ± 0.2 s and the mean amplitude was 8.9 ± 2.2 ml/min/100 g. On the other hand, a spike-like artefact occurred more frequently in hand skin blood flow (57 times). The mean duration of the artefacts was 0.8 ± 0.1 s and the mean amplitude was 3.8 ± 0.5 ml/min/100 g. Taking the whole observation period into account, a distortion of forehead and hand skin blood flow data by removal of the artefacts was negligible (less than 0.16%). Thus the present data collection involved no substantial noise due to facial expression and/or hand movement during the emotional challenges.

### Data and statistical analyses

The mean cardiovascular variables before and during emotional interventions for 2 min were averaged over the three trials of a given emotional intervention and further averaged in 18 subjects. The group-averaged cardiovascular data were analysed by two way repeated measures ANOVA with the factors of time period (before vs. during) and emotional intervention (comedy, horror, and landscape). If significant differences in the main effects and interaction were obtained, a Student-Newman-Keuls posthoc test was performed for all pairwise comparisons.

Regarding the prefrontal NIRS, all multichannel Oxy-Hb and Deoxy-Hb signals were stored to a computer of the FOIRE-3000 at a sampling frequency of 7.7 Hz. In off-line condition, the raw NIRS data were resampled sequentially as an average every one second without any filtering procedure. At each of the 22 prefrontal locations, the resampled data were averaged over the three trials of each emotional intervention and further averaged in 18 subjects (as shown in Fig. 4). The group-averaged time course data were analysed by a Friedman ANOVA on ranks. If a significant difference in the main effect of time was obtained, a Dunn’s posthoc test was performed to detect a difference in the mean values at a given time from the baseline. The latency was defined as the shortest time interval from the onset of emotional intervention to the significant changes. Next, using the same time course data, the mean Oxy-Hb response for 2 min was calculated for a given emotional intervention and averaged in 18 subjects. At each prefrontal location, a difference in the average Oxy-Hb response from the baseline was examined by one sample t-test. The dataset of the prefrontal Oxy-Hb responses was analysed by two way repeated measures ANOVA with the factors of the prefrontal location and emotional intervention (comedy, landscape, and horror). If significant differences in the main effects and interaction were obtained, a Student-Newman-Keuls posthoc test was performed for all pairwise comparisons in the Oxy-Hb response. Assuming that landscape intervention was considered a neutral emotional condition (as judged from the subjective data in Fig. 1) and involved non-emotional audiovisual stimulation, the prefrontal Oxy-Hb responses were subtracted between the comedy and landscape condition and between the horror and landscape condition (as shown in Fig. 4B), to disregard an influence of non-emotional audiovisual stimulation. The subtracted Oxy-Hb responses were similarly analysed by one sample t-test and two-way repeated measures ANOVA.

At a given NIRS channel, the time course data of the changes in prefrontal Oxy-Hb, skin blood flow (cheek, forehead, and hand), and MAP during each trial of comedy stimulation were averaged in 18 subjects (as shown in Fig. 5A). To examine to what extent the activity in the PFC is correlated with the changes in peripheral circulatory variables, a Spearman correlation analysis between the prefrontal Oxy-Hb response and peripheral circulatory variables was performed using the time course data. The squares values (*γ*^2^) of Spearman’s rank correlation coefficients in relation to peripheral variables (facial and hand skin blood flow and MAP) were obtained for each trial of comedy stimulation and for each of the 22 prefrontal locations. The *γ*^2^ value was considered as a dependent variable, while the NIRS channel location and peripheral parameters (facial and hand skin blood flow and MAP) were considered as independent variables. By comparing repeated measurements among the individual trials of comedy stimulation, the dataset of the *γ*^2^ values were analysed by two way repeated measures ANOVA with the factors of prefrontal location and peripheral parameter. If significant differences in the main effects and interaction were detected, all pairwise multiple comparisons were performed with the Student-Newman-Keuls posthoc test.

Actually, the reduction in the prefrontal Oxy-Hb correlated to the decrease in forehead skin blood flow, suggesting that the prefrontal NIRS responses might simply reflect a fall in extracranial vascular blood flow within the illuminated tissue area. To examine the possibility, the changes in forehead skin blood flow were compared with the simultaneous changes in the prefrontal Oxy-Hb during conversation using the identical subjects; because we know that conversation greatly increases prefrontal oxygenation but do not always elicit emotional changes. Conversation was made whenever the subjective ratings of pleasantness and consciousness were asked. The subjective ratings of pleasantness and consciousness were analysed by two way repeated measures ANOVA and posthoc Student-Newman-Keuls test with the main effects of trial number and emotional intervention (comedy, horror, and landscape). The overall relationship between the subjective ratings of pleasantness and consciousness was assessed by a Spearman’s rank order correlation method. A level of statistical significance was defined at *P* < 0.05 in all cases. All statistical analyses were performed using SigmaPlot^®^ version 13.0 (Systat Software, San Jose, CA). All variables are expressed as means ± S. E. M.

## Electronic supplementary material

Supplementary Figures
