# Supplementary material for: Deactivation of the prefrontal cortex during exposure to pleasantly-charged emotional challenge
Source: Sci Rep. 2018 Sep 28;8:14540. doi: 10.1038/s41598-018-32752-0 (PMC6162331; doi:10.1038/s41598-018-32752-0)
Supplement: Supplementary file 1 — Supplementary Figures [file 41598_2018_32752_MOESM1_ESM.pdf]

Supplementary information

Deactivation of the prefrontal cortex during exposure to pleasantly-charged emotional challenge

by

Kanji Matsukawa*[1], Ryota Asahara[1], Miho Yoshikawa[1], and Kana Endo[1]

Department of Integrative Physiology[1], Graduate School of Biomedical and Health Sciences, Hiroshima University, 1-2-3 Kasumi, Minami-ku, Hiroshima 734-8551, Japan

Legends of Supplementary Figures

Supplementary Figure S1 The dynamic changes in concentrations of prefrontal oxygenated-haemoglobin (Oxy-Hb, with red lines) and deoxygenated-haemoglobin (Deoxy-Hb, with blue lines) are represented over the 22 NIRS channels during emotionally-charged challenges in the same subject. The changes in prefrontal Oxy- and Deoxy-Hb are superimposed in three consecutive trials of each emotional intervention (comedy, landscape, and horror).

Supplementary Figure S2 The anatomical locations of all near-infrared spectroscopy (NIRS) probes, estimated by a three-dimensional digitizer, are summarised. BA, Brodmann's area. The anatomical brain region and BA corresponding to the location of a NIRS probe are suggested with their occurrence probability (mean ±S. E. M.).

Supplementary Figure S1A

A. Comedy

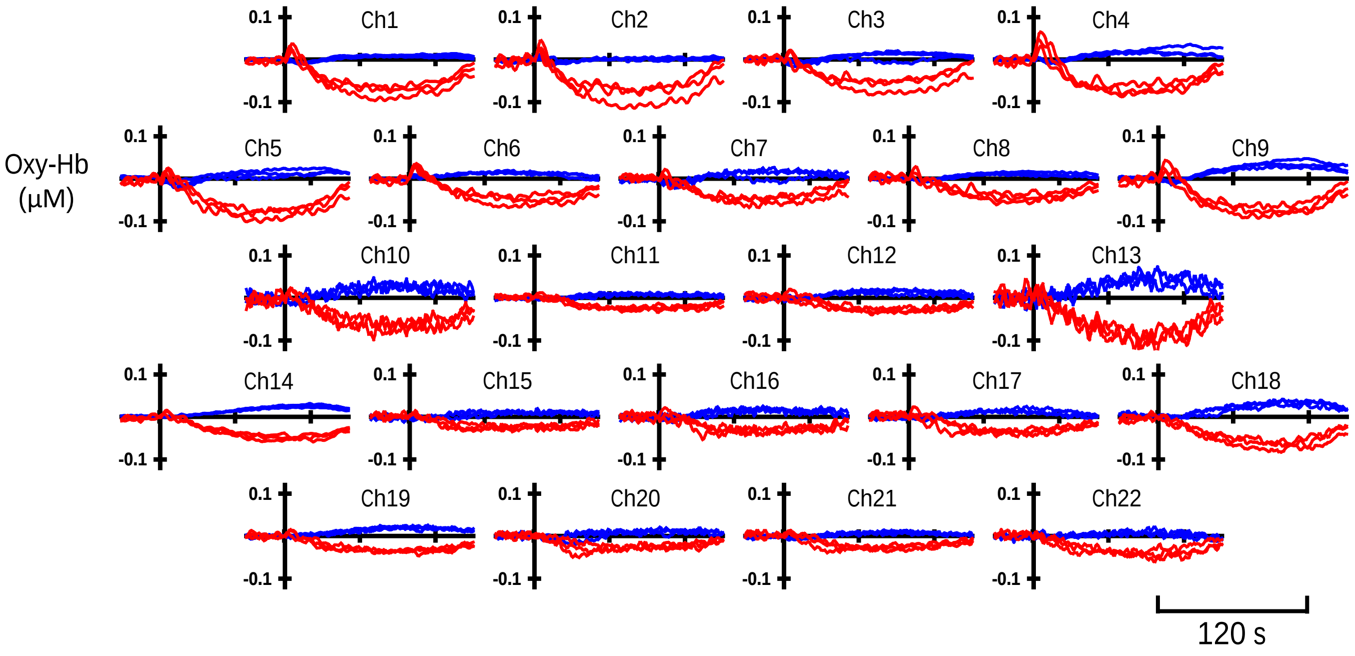

The dynamic changes in concentrations of prefrontal oxygenated-haemoglobin (Oxy-Hb, with red lines) and deoxygenated-haemoglobin (Deoxy-Hb, with blue lines) are represented over the 22 NIRS channels during exposure to the comedy movie in the same subject. The changes in prefrontal Oxy- and Deoxy-Hb are superimposed in three consecutive trials of comedy stimulation.

Supplementary Figure S1B

B. Landscape

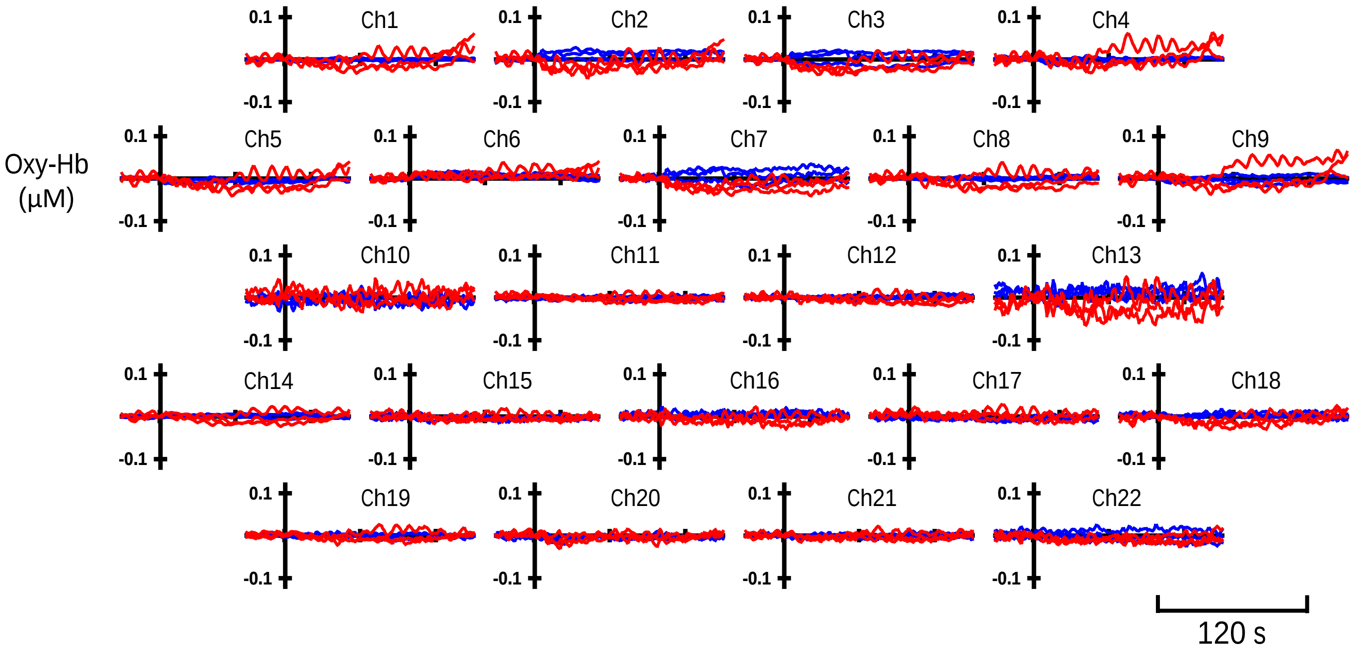

The dynamic changes in concentrations of prefrontal oxygenated-haemoglobin (Oxy-Hb, with red lines) and deoxygenated-haemoglobin (Deoxy-Hb, with blue lines) are represented over the 22 NIRS channels during exposure to the landscape movie in the same subject. The changes in prefrontal Oxy- and Deoxy-Hb are superimposed in three consecutive trials of landscape stimulation.

Supplementary Figure S1C

C. Horror

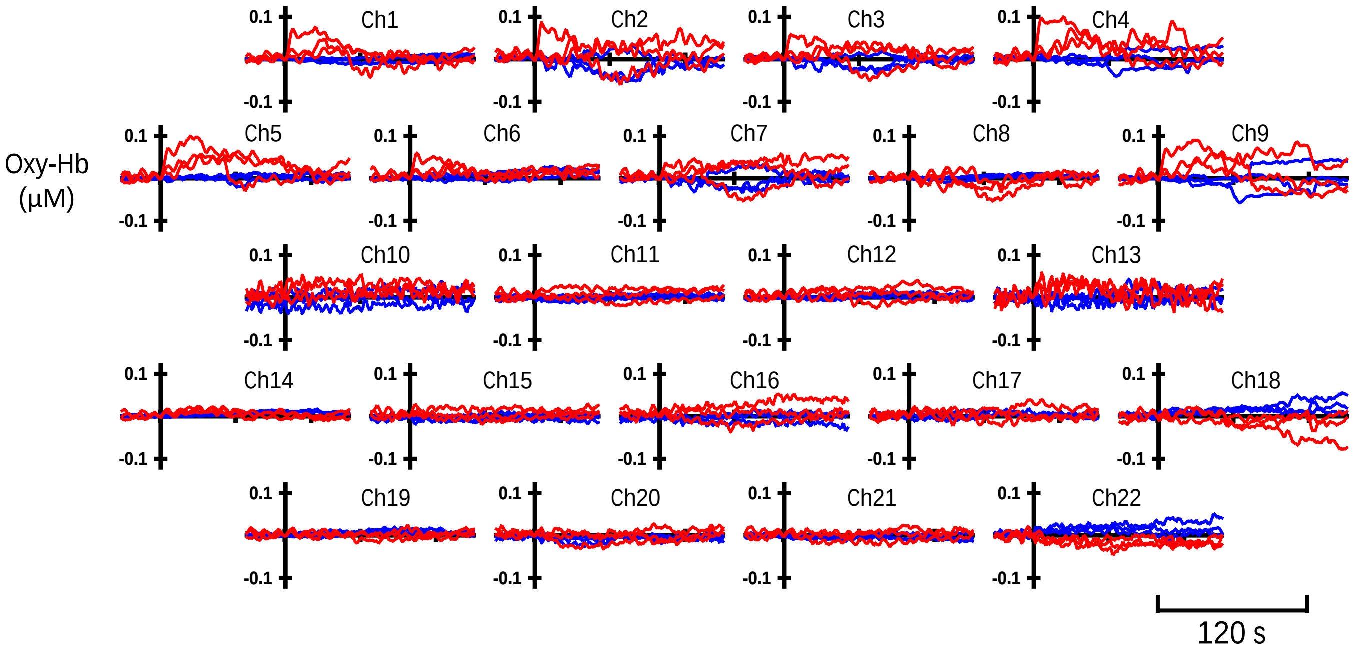

The dynamic changes in concentrations of prefrontal oxygenated-haemoglobin (Oxy-Hb, with red lines) and deoxygenated-haemoglobin (Deoxy-Hb, with blue lines) are represented over the 22 NIRS channels during exposure to the horror movie in the same subject. The changes in prefrontal Oxy- and Deoxy-Hb are superimposed in three consecutive trials of horror stimulation.

Supplementary Figure S2

NIRS-Channels	Anatomical cortical region	BA area	Probability (%)
Ch1	Frontopolar area<br>Orbitofrontal area	BA10<br>BA11	65±5<br>21±4
Ch2	Frontopolar area<br>Orbitofrontal area	BA10<br>BA11	87±6<br>13±6
Ch3	Frontopolar area<br>Orbitofrontal area	BA10<br>BA11	87±5<br>13±5
Ch4	Frontopolar area<br>Orbitofrontal area	BA10<br>BA11	64±6<br>26±6
Ch5	Dorsolateral prefrontal cortex<br>Frontopolar area<br>pars triangularis Broca's area	BA46<br>BA10<br>BA45	64±3<br>18±5<br>17±5
Ch6	Frontopolar area	BA10	88±5
Ch7	Frontopolar area	BA10	95±2
Ch8	Frontopolar area	BA10	86±3
Ch9	Dorsolateral prefrontal cortex<br>pars triangularis Broca's area	BA46<br>BA45	69±4<br>25±6
Ch10	Dorsolateral prefrontal cortex<br>Frontopolar area	BA46<br>BA10	74±4<br>16±5
Ch11	Frontopolar area<br>Dorsolateral prefrontal cortex	BA10<br>BA9	73±8<br>26±8
Ch12	Frontopolar area<br>Dorsolateral prefrontal cortex	BA10<br>BA9	69±9<br>23±8
Ch13	Dorsolateral prefrontal cortex<br>Frontopolar area<br>pars triangularis Broca's area	BA46<br>BA10<br>BA45	69±5<br>16±6<br>13±3
Ch14	pars triangularis Broca's area<br>Dorsolateral prefrontal cortex	BA45<br>BA46	55±8<br>38±7
Ch15	Dorsolateral prefrontal cortex<br>Dorsolateral prefrontal cortex<br>Frontopolar area	BA9<br>BA46<br>BA10	63±9<br>24±8<br>14±5
Ch16	Dorsolateral prefrontal cortex<br>Frontopolar area	BA9<br>BA10	68±6<br>29±7
Ch17	Dorsolateral prefrontal cortex<br>Dorsolateral prefrontal cortex<br>Frontopolar area	BA9<br>BA46<br>BA10	52±8<br>27±5<br>21±7
Ch18	pars triangularis Broca's area<br>Dorsolateral prefrontal cortex	BA45<br>BA46	75±5<br>22±5
Ch19	Dorsolateral prefrontal cortex<br>Dorsolateral prefrontal cortex	BA9<br>BA46	61±8<br>30±6
Ch20	Dorsolateral prefrontal cortex<br>Includes Frontal eye fields	BA9<br>BA8	84±6<br>15±6
Ch21	Dorsolateral prefrontal cortex<br>Includes Frontal eye fields	BA9<br>BA8	84±6<br>15±7
Ch22	Dorsolateral prefrontal cortex<br>Dorsolateral prefrontal cortex	BA9<br>BA46	57±6<br>35±6

The anatomical locations of all near-infrared spectroscopy (NIRS) probes, estimated by a magnetic three-dimensional digitizer, are summarised. BA, Brodmann's area. The anatomical brain region and BA corresponding to the location of a NIRS probe are suggested with their occurrence probability (mean ±S. E. M.).
